# Carnivory in the larvae of *Drosophila melanogaster* and other *Drosophila* species

**DOI:** 10.1038/s41598-018-33906-w

**Published:** 2018-10-19

**Authors:** Daxiang Yang

**Affiliations:** Department of Zoology and Animal Physiology, College of Biological Sciences, China Agricultural University, 100193 Beijing, China

## Abstract

*Drosophila melanogaster* is widely used as a model organism for biological investigations, and food is a major aspect of its ecology and evolutionary biology. Previous studies have shown that this insect can use fruits, yeasts and insect carcasses as its food sources. In this study, we demonstrate that this species is an omnivore, that its larvae can exploit not only fruits and yeast but also foods of animal origin (FAOs), and that larvae consume adult carcasses regularly. FAO-fed larvae develop into adulthood within a normal developmental time frame without the help of microbes. Yeast foods are better for *Drosophila* development than are foods of plant origin (FPOs) or FAO because in yeast foods, more eggs complete their life cycle, and the body size of emerged flies is much greater. Flies can use a mixture of yeast-FAO, which significantly boosts female fertility. Larvae digest FAOs externally. Larval *D*. *virilis*, *D*. *hydei*, and *D*. *simulans* are also omnivorous and demonstrate the same feeding habits as larval *D*. *melanogaster*. These findings prompt us to reconsider previous conclusions about the original adaptations of *D*. *melanogaster* and other *Drosophila* species and have direct implications for diet-related studies using *Drosophila* as a model organism.

## Introduction

Food affects animal well-being, behavior, physiology, lifespan^1^, and reproductive traits^2^ and defines the ecological niche, geographic distribution and abundance^3^, and temporal activity pattern of animals^4^. When physiological or environmental conditions change, animals shift their food preferences to cope with fluctuations in food availability^5^. Some genes are involved in animal food preference^6,7^, and genetic changes are responsible for shifts in host plant or diet. Food is a major aspect of animal ecology, and understanding the diets that an animal can exploit and the interactions between an animal and its food has broad implications across ecology, genetics and evolutionary biology^8^. Determining the possible diets of animals that are used in studies of aging, nutrition, and diet-induced pathophysiological mechanisms^9^ is an important step.

*Drosophila melanogaster* has an Afrotropical origin and originally used sixteen native plant species as its food sources.^3^ It can be bred in a laboratory and fed many domesticated fruits or media made of domesticated fruits and agar^10^, which suggests that it eats fruit. However, starting in the 1950s, evidence^8,11–14^ began to accumulate that suggested that the major food source of both the larvae and adults of this species was yeasts associated with plant fermentation. Adult *Drosophila* are attracted to food by the odor of yeast^12^; with the help of the gustatory and olfaction systems, females estimate the nutritional value and potential toxicity of foods and choose a suitable oviposition site^15–17^. *Drosophila* larvae usually associate in clusters to dig more effectively into food^18^ and egest digestive enzymes that are supposed to be produced by massive salivary glands onto the substrate to perform “social digestion”^19^. A total of 349 putative digestive enzyme genes have been identified in the *Drosophila* genome^20^. Genes related to taste perception, food intake, food preference, foraging and locomotion have been reported^5,21,22^.

However, many surveys and studies suggest that the diets of *D*. *melanogaster* include many more foods. *D*. *melanogaster* has been found in products such as canned fruit and pickles, human excrement^23^, human nasal cavities^24^, and carcasses of spiders or insects^19,25^, and it can breed successfully in the wild on dead Lepidoptera caterpillars^26^. When placed under nutritional stress, both larval and adult *Drosophila* are able to consume their own kind^25,27^. Here, we report that *D*. *melanogaster* larvae are able to exploit a variety of foods of animal origin (FAO), complete their entire development within a normal time frame without the help of microorganisms, and consume adult carcasses regularly. They egest proteinase and lipase onto food surfaces and perform extraoral digestion. Other species, such as *D*. *simulans*, *D*. *virilis* and *D*. *hydei*, are also omnivorous insects.

## Results

### Adults feed and oviposit on FAOs

**Figure 1:**
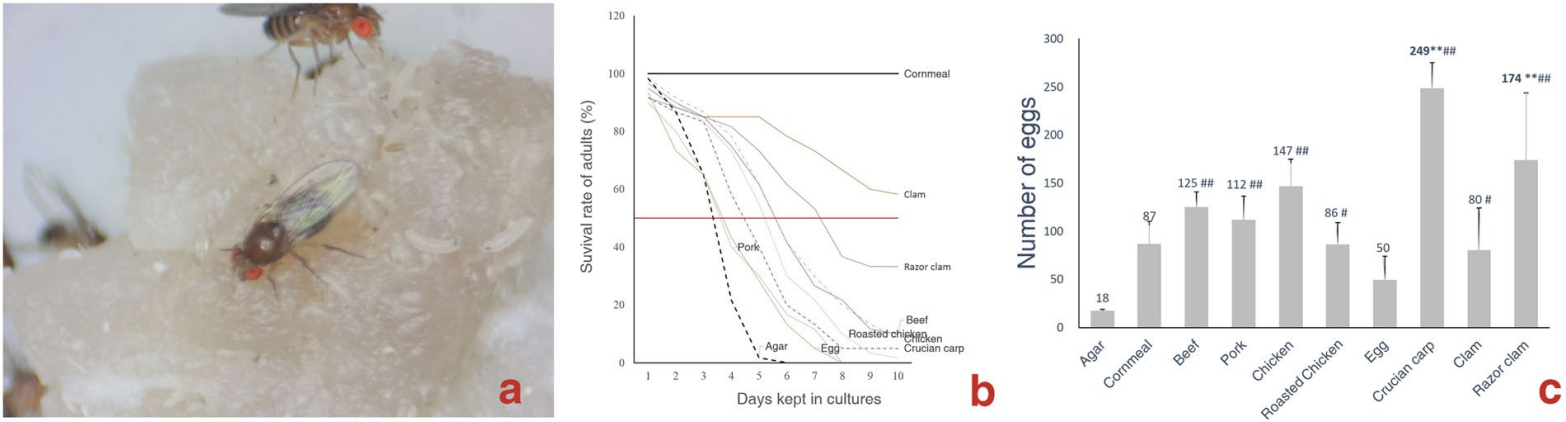
Adult *D*. *melanogaster* forage and lay eggs on FAOs. (**a**) Adults and larvae feed on pork. (**b**) The cumulative survival rates of the flies maintained on FAOs. Half of the adults died within 6 days, and nearly 90% of flies died within 10 days, except those in clams and razor clams. (**c**) Number of eggs laid on agar gel, cornmeal medium and FAOs. ^#, ##^ indicate significantly (P < 0.05)/very significantly (P < 0.01) more than those in agar gel; ** indicate significantly (P < 0.01) more than those in cornmeal media.

All the adult *D*. *melanogaster* died within 48 hours without food and water, but they survived for five days when fed pure agar gel. When FAOs were placed on the agar gel, the adults foraged on them and survived longer (Fig. 1a,b), but more than 90% of the individuals died within 10 days. The cumulative survival rates of the flies fed FAOs were not significantly (*χ*^2^ = 8.853, P = 0.355) higher than those of flies fed agar gel and were significantly lower than those fed cornmeal medium (P = 0.000).

Females oviposited on or within the vicinity of FAOs. The mean cumulative number of eggs laid on FAOs within 72 hours ranged from 50 to 249 (Fig. 1c), which was significantly (P < 0.05 or P < 0.01) greater than that laid on pure agar gel and not greater than that laid on cornmeal medium patches. We conclude that although the adults failed to consume sufficient nutrients from FAOs for their survival, as adult flies intermittently ingest liquid via their proboscis^20^, the adults evaluated and selected FAOs for their offspring. The hatchability of eggs laid on agar gel, FAOs and cornmeal medium patches ranged from 0.87 to 0.98, with a mean of 92%, and there was no significant difference among these values.

### Larvae maintained on FAOs developed into adulthood with a normal life cycle

**Figure 2:**
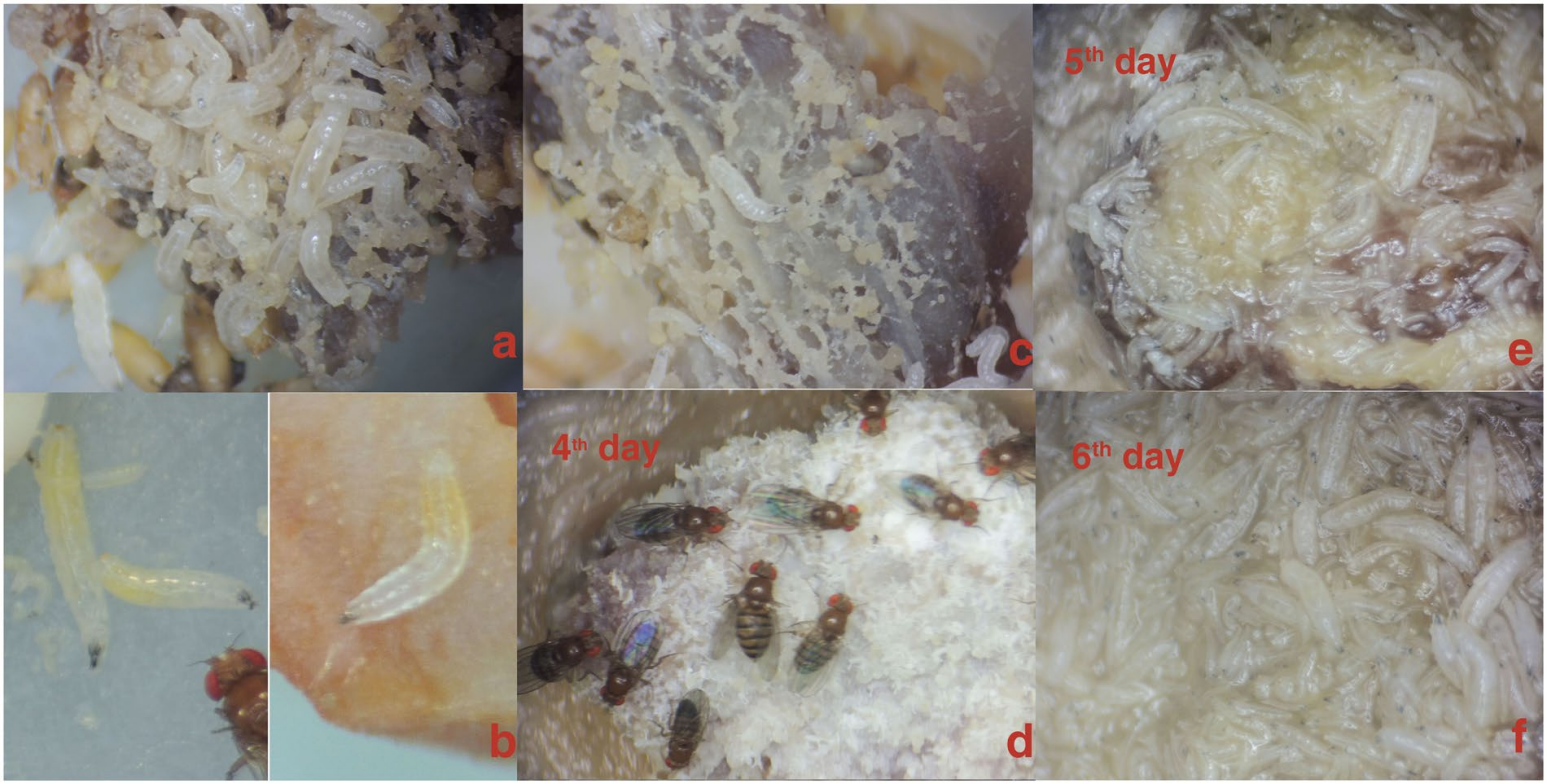
*D*. *melanogaster* larvae feed on FAOs. (**a**) Larvae associate in clusters and feed underneath beef (the beef was turned upside down to take the picture in **a** and **c**). (**b**) The yellow yolk (left) and the orange food dye (right) can be seen in the guts of the larvae. (**c**) The “canals and ditches” dug underneath beef by foraging larvae. (**d**–**f**) The adults fed and laid eggs on moldy beef on the 4^th^ day, and on the 5^th^ day, the mold was removed by a cohort of larvae. The meat was moistened, liquefied and eaten by the larvae on the 6^th^ day.

*D*. *melanogaster* larvae may forage on a food surface (Fig. 1a), but they tend to associate in clusters and forage underneath foods (Fig. 2a). This behavior may help avoid a predator’s attack when they are foraging^28^. Moreover, the top of food that is exposed to air would dry up and become hard, making it difficult for larvae to eat. In some cases, the foods ingested by larvae can be seen directly in their gut (Fig. 2b), allowing researchers to determine whether the food ingested by larvae is derived from FAOs. The mouth hooks of larvae are strong enough to excavate “tunnels” through FAOs or dig “canals and ditches” on the surface (Fig. 2c). *Drosophila* larvae the consumer of microbes, are able to exploit decaying (which stink) or moldy FAOs and grow well. In this study, when larval population sizes were large enough, it was easy to see that the FAOs were first moistened and after a few days were liquefied and then eaten (Fig. 2d–f). All this information suggests that both mechanical and biochemical processes are involved in larval digestion of FAOs.

The adult flies emerged on the 10^th^ day at 25 °C, as did those that fed on cornmeal medium. Sterile eggs fed on axenic FAOs could also develop to adulthood within 10 days. This result indicated that larvae can use FAOs without the help of microorganisms. As previously mentioned, more than 90% of the eggs placed on FAOs hatched into larvae, but only a small proportion of them completed their entire life cycle. As shown in Table 1, the ratio of adults that emerged from eggs placed on cornmeal medium was 55.3 ± 6.1%, but when the eggs were placed on FAOs, these ratios were less than 20%, and only 1 adult emerged from the 100 eggs placed on the clam. In comparison, eggs were placed on some foods of plant origin (FPOs). The mean ratios of adults that emerged from eggs placed on banana and mango were also less than 20%, and no adults emerged when eggs were placed on peach, apple, pear or muskmelon. The ratios of adults that emerged from eggs placed on FAOs or FPOs were not significantly different, and they were all significantly (P = 0.01) less than the ratio of adults that emerged from. the cornmeal medium, suggesting that for *Drosophila* development, FPOs are not better than FAOs and that yeast food is the best.

**Table 1:** The ratio of adults that emerged from eggs placed on different foods and adult size.

Foods	Ratio of adults emerged/100 eggs(%)	Tukey Grouping		Adult size (30 adults/mg)	
		0.05	0.01	female	male
Cornmeal (CK)	55.3 ± 6.1	a	A	39.6	23.8
Mango	18 ± 9.5	b	B	13.8	8.55
Chicken	17.3 ± 7.2	b	B		
Pork	15.7 ± 1.5	b	B		
Banana	15.3 ± 5.9	b	B		
Beef	14 ± 1	b	B	17.6	13.6
Peach	0				
Apple	0				
Pear	0				
Muskmelon	0				
Clam	1 ± 1				
Roasted chicken	—			19.1	16.2
Razor clam	—			20.1	15.5

The adults that emerged from FAOs were markedly smaller in size than those bred from the cornmeal medium (Table 1). The cornmeal medium-fed adult females were almost two times heavier than those bred from FAOs or FPOs. When FAO-fed light-weight flies were transferred to cornmeal medium, they gained weight. For example, when beef-fed flies were transferred to cornmeal medium for 4 days, female size increased from 17.6 mg to 28.3 mg and male size from 13.6 mg to 16.6 mg. These FAO-fed flies were fertile (Table 2). It is interesting to note that the progeny of the FAO-fed. flies were significantly (P = 0.05 or P = 0.01) greater in number but significantly (P = 0.05 or P = 0.01) lighter in weight than the progeny of the cornmeal medium-fed flies.

**Table 2:** Progeny number and progeny size of the FAO-fed flies.

	Progeny number	Progeny size (30 adults/mg)	
		Female	Male
Cornmeal (CK)	113.5	39.7	23.7
Beef	148*	33.4*^,^**	20.9*
Pork	165*^,^**	33.2*^,^**	20.3*
Chicken	163.5*^,^**	33.4*^,^**	23.4

*Drosophila* larvae perform social digestion; thus, increasing the size of the larval population will increase the amount of digestive enzymes on a food surface and make digestion easier. This scenario raises a question: does the larval population size increase in proportion to the number of adults that emerge? To answer this question, 20, 40, 120, 300 and 500 eggs were placed on 2 g of beef, and the proportions of adults that emerged were 0.48 ± 0.28, 0.46 ± 0.12, 0.19 ± 0.16, 0.06 ± 0.07 and 0.21 ± 0.16, respectively. It seems that the number of adults that emerged was stochastic.

**Figure 3:**
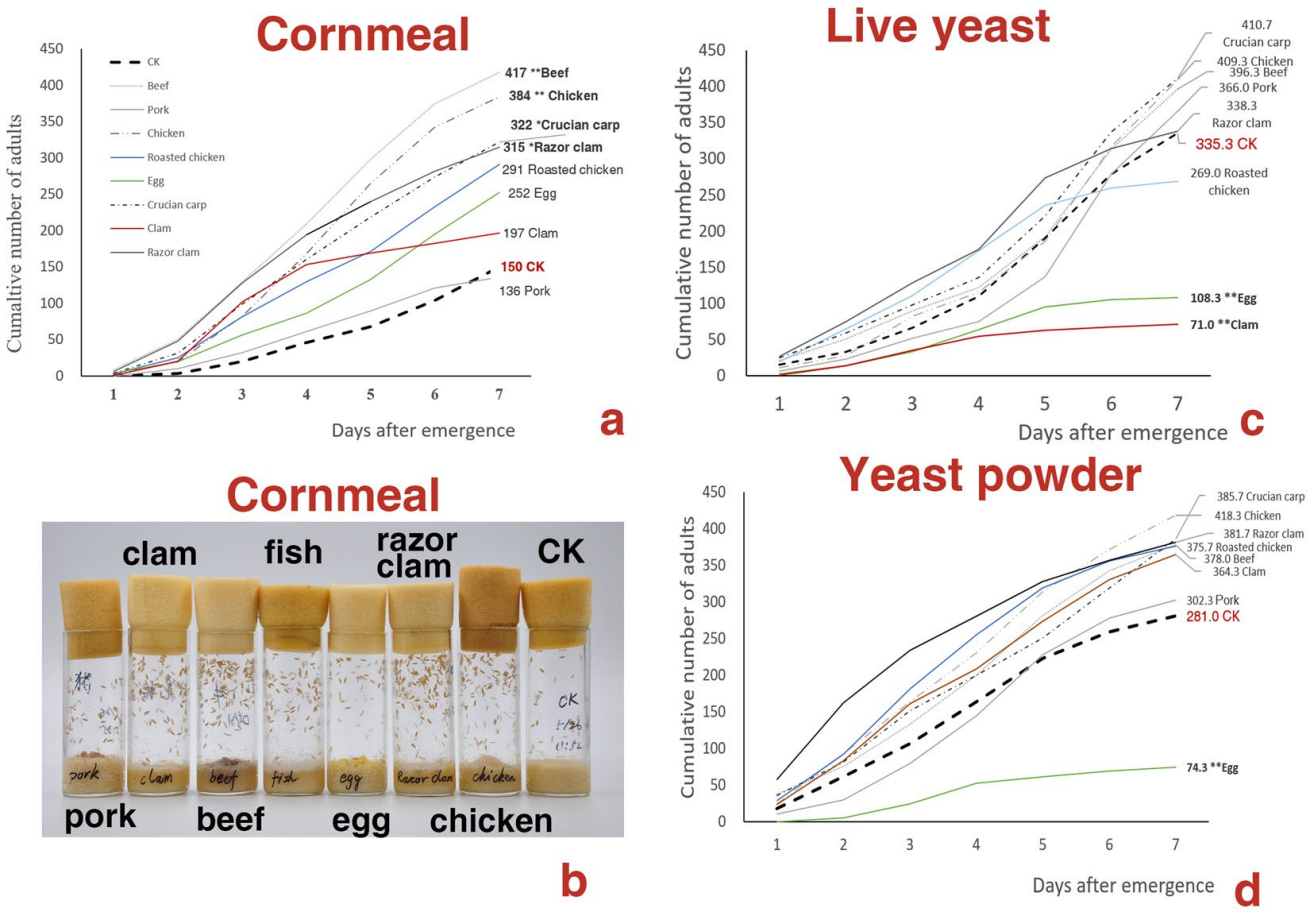
Enhanced effect of combined FAO-yeast foods on the fertility of *D*. *melanogaster*. (**a**,**b**) The numbers of adults that emerged from combined cornmeal-FAO foods were higher than those that emerged from the control food, which can be seen directly in the number of pupa in (**b**). (**c**) The numbers of adults that emerged from combined yeast-FAO foods were higher than those that emerged from the control food. However, it is interesting to note that when chicken eggs and clams were placed on a yeast medium, the numbers of adults that emerged were less than those that emerged from the control food, and the reason for this result is still unknown. *, ** indicate

Yeasts are considered a major food source for the saprophagous *Drosophila* in both the adult and larval stages^8^; however, under some nutritional conditions, such as domestic garbage, FAOs and fermented fruits may mix together. In this case, will flies choose a single food or both foods? To see the combined effect of FAO-yeast foods on adult and larval *Drosophila*, 0.8 g FAOs was placed on yeast media. The FAOs covered only part of the yeast media so that adults were able to choose freely between both food sources. Adults were found feeding and depositing a large amount of eggs continuously on the FAOs, and the juveniles associated in clusters and fed on FAOs greedily (Fig. 2d–f, Movies S1 and S2) until the FAOs were consumed entirely. The mean daily collections of emerged flies from combined FAO-yeast diets were generally much larger than those from those from the control yeast foods (Fig. 3a–d). Adult and larval

*Drosophila* chose both FAOs and yeast foods when these foods were presented simultaneously and mixed together; these new food resources significantly increased the number of eggs laid and the number of flies that emerged, demonstrating an enhanced effect. However, when FAOs and yeast foods were separated, both adult egg laying and larvae foraging behaviors were altered, which will be discussed in the next section.

### Larvae and adults prefer yeast food over FAOs

**Figure 4:**
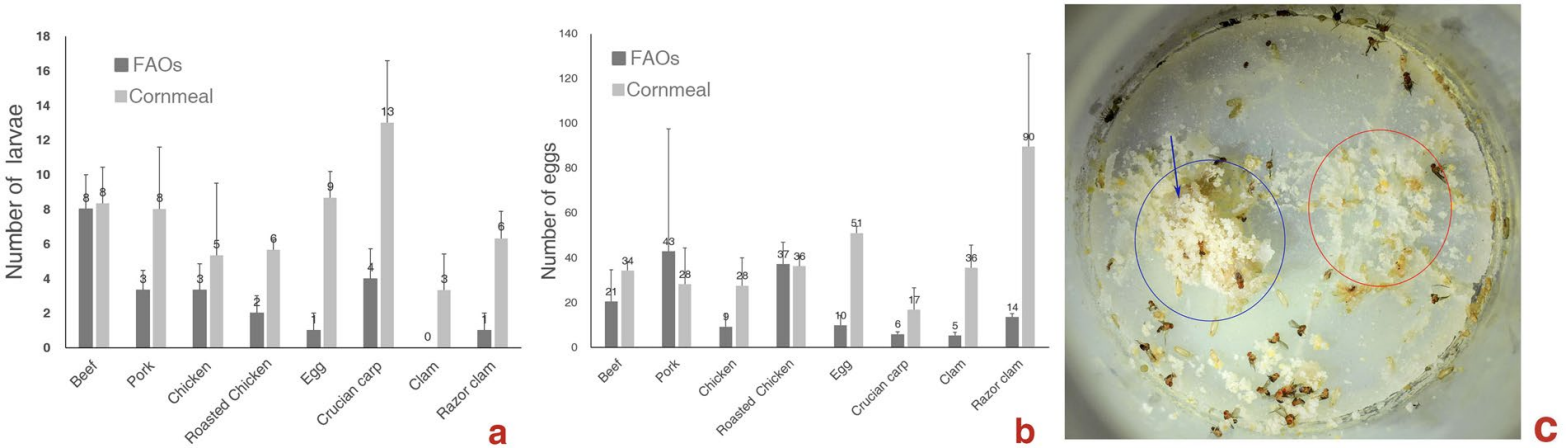
Food preferences of larval and adult *D*. *melanogaster*. (**a**) Numbers of larvae aggregated around foods. (**b**) Numbers of eggs laid on foods. (**c**) On the 15th day, the cornmeal patch (red circle) was consumed entirely, and the pork patch (blue circle) remained.

When a cornmeal patch and an FAO of the same weight were available simultaneously but were placed apart and larval and adult *D*. *melanogaster* were allowed to choose freely between two adjacent food patches, they showed a more biased preference for yeast food. In each cornmeal patch-FAO set, more larvae aggregated around the cornmeal patch, and more eggs were laid on the cornmeal patch (Fig. 4a–c). The larval choices of diet (*t* = 6.044, P = 0.000) and egg-laying site (*t* = 2.232, P = 0.004) were significantly different from chance. When the egg-laying preference tests were finished, the larvae in the pork, beef and cornmeal medium patches together with their foods were placed back to where they were and maintained at 25 °C for further observation. When the larvae were allowed to self-select a favorable nutrient mix^29,30^, they no longer clustered around the pork or beef and consumed them entirely before they turn to yeast foods, as shown in Fig. 2e,f, but they consumed the cornmeal medium patch first, and some FAOs remained (Fig. 4d). First, these results indicate that both larval and adult *Drosophila* prefer yeast food over FAOs; second, these results indicate that although larval and adult *Drosophila* do not totally reject FAOs, the forage and egg laying behaviors of adults and the forage behavior of larvae are strongly affected by the distance between patchily distributed foods.

### Adult carcasses are a regular food for larvae

*D*. *melanogaster* is the only cannibalistic species reported in the genus *Drosophilidae*^25,31^. Larvae raised on several nutrient-rich media under standard laboratory conditions have always been found to consume conspecific adult carcasses (Movies S3 and S4). These media include holidic medium^32^, cornmeal medium, yeast medium and FAOs. Adult heads, legs, wings, and cuticle pieces can always be found in such cultures.

To exclude the possibility that the cannibalistic behavior was induced by starvation or by overcrowdedness, 15–20 cornmeal-fed mixed-age larvae were randomly selected and placed into a fresh cornmeal culture, and they were left to forage freely for 3–4 hours before putting 4–5 intact, frost-killed adult *D*. *melanogaster*, *D*. *simulans*, or *D*. *hydei* into the same culture vials. Then, the carcasses were closely observed under a stereomicroscope every 12 hours. Pierced carcasses were found in vials within 24 hours, and all carcasses were burrowed within 48 hours. The larvae first broke the cuticle of the ventral side of the abdomen of a carcass and then consumed the viscera, followed by the other parts of the body. These results suggest that larval consumption of conspecific/nonconspecific adult carcasses was not induced by starvation or overcrowdedness. However, the larvae that fed exclusively on adult carcasses all died before pupation. A possible biological function of this feeding habit is helping larvae survive extreme nutritional conditions before they find new food sources.

However, larval consumption of conspecific eggs or pupa is not regular behavior. No egg remains were found in the vicinity of the dismembered adult bodies when the nearly 100 cornmeal/FAO/combined yeast-FAO diet cultures were closely examined. The eggs in three combined yeast-FAO diet cultures thickly crowded with larvae were observed for 3 hours at a 30-minute interval, and no larval aggregation or egg consumption was found. The locations of 30 eggs in six FAO cultures were carefully marked and observed for 72 hours at 12-hour intervals. The eggs that remained unhatched were found intact at their original locations, which indicated that larval consumption of eggs is not a regular behavior. In addition, larval consumption of pupae was not found in this study. Eggs and pupae ensure the continuation of a species, as they are the future of a species. It would be destructive to a species if the eggs or pupae were consumed readily by their older siblings.

### Some *Drosophila* species are also omnivorous insects

**Figure 5:**
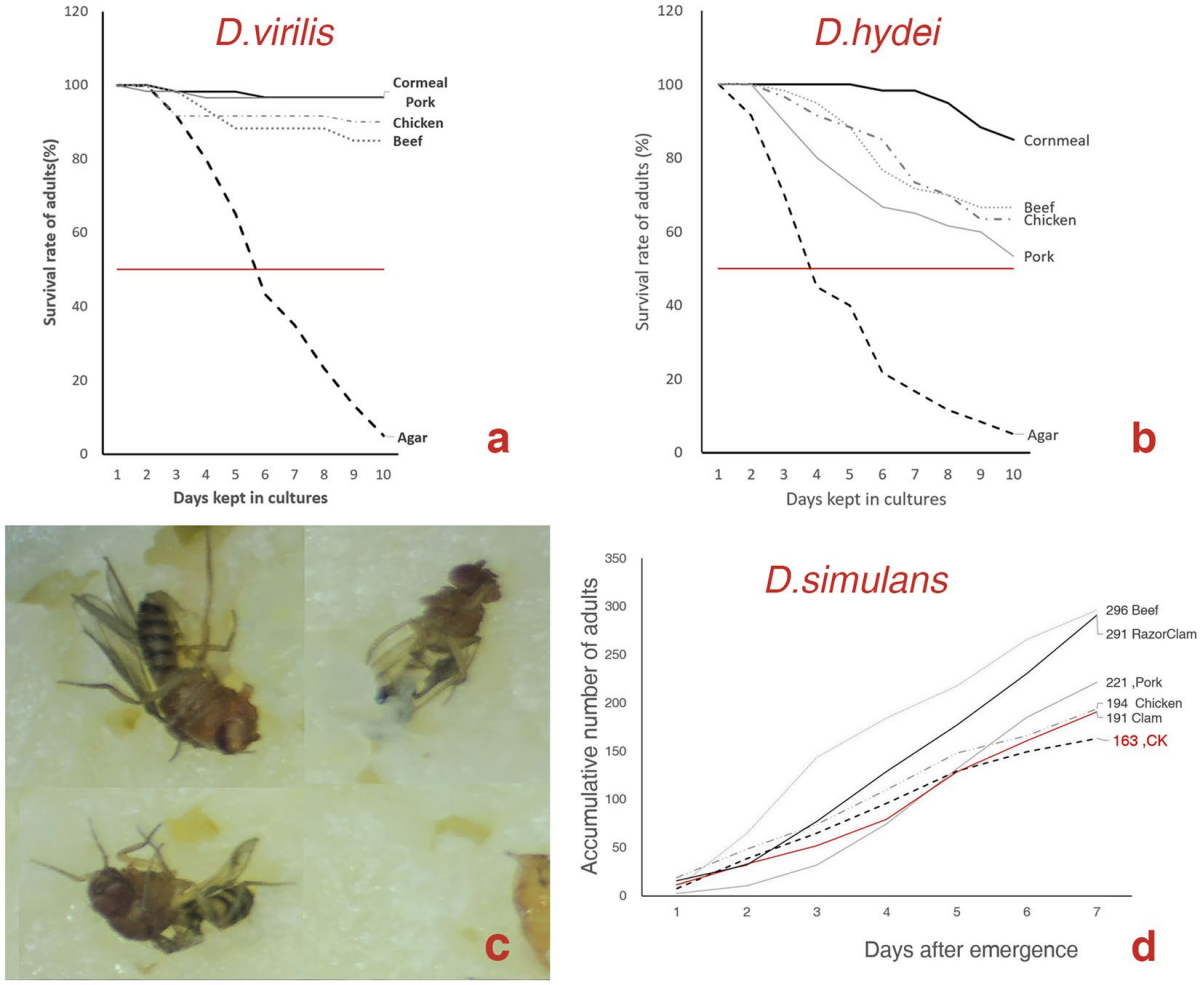
Omnivorous feeding habits of *D*.*virilis*, *D*.*hydei* and *D*. *simulans*. (**a**,**b**) The cumulative survival rates of the *D*.*virilis* (**a**) and *D*. *hydei* (**b**) maintained on FAOs. (**c**) The carcasses of *D*. *melanogaster* were pierced by 15 well-fed *D*. *simulans* larvae within 36 hours. (**d**) The cumulative number of emerged adults in the cornmeal medium (CK) and cornmeal-FAO diets. The animal diet patches (0.8 g per patch) were placed on the cornmeal media. The cornmeal-FAO diets boosted the female fertility of *D*. *simulans*.

Adult *D*. *virilis* and *D*. *hydei* can survive as long as 10 days when fed pure agar gel; this survival period is 2 times longer than that of adult *D*. *melanogaster* (Fig. 5a,b). On the 10^th^ day, more than 85% of adult *D*. *virilis* and 53% of *D*. *hydei* were still alive. The cumulative survival rates of the flies that were fed FAOs were significantly (*D*. *virilis*: *χ*^2^ = 10.866, P = 0.012; *D*. *hydei*: *χ*^2^ = 9.642, P = 0.022) higher than those fed agar gel, but they were generally significantly lower than those that fed cornmeal medium (*D*. *virilis*: *χ*^2^ = 9.819, P = 0.02; *D*. *hydei*: *χ*^2^ = 9.053, P = 0.029). The adults of *D*. *virilis* and *D*. *hydei* fed and laid eggs on FAOs. The life cycle of *D*. *virilis* that was fed FAOs was the same as that fed cornmeal medium, but the adults that emerged were smaller in size (beef fed: 30 mg♀/28 mg♂; cornmeal fed: 50 mg♀/47.2 mg♂). The *D*. *hydei* pupae were found on the 10^th^ day in the cornmeal medium, while even on the 20^th^ day, no pupae were found in the FAO culture vials. In a previous report, *D*. *hydei* was found to breed on beef with a longer developmental time^26^, but in this study, it was unclear whether the larvae in these vials could have continued their development and completed their entire life cycle because, starting on the 20^th^ day, they died off as the food dried up and became hard.

Next, we tested the effects of the combined cornmeal-FAO diet by feed the adults combined cornmeal-beef/chicken. It is interesting to note that the adults of *D*. *virilis* seemed reluctant to lay eggs on FAOs and deposited many more eggs on the cornmeal medium. Their larvae also seemed to be strict vegetarians at first but switched rather abruptly into greedy carnivores a few days later. The females deposited eggs on the conspecific adult carcasses, and this behavior was not found in the other species in this study. In the cornmeal-FAO combined diets, both adult and larval *D*. *hydei* preferred the FAOs, the females deposited many eggs on the FAOs, the larvae clustered around the FAOs and devoured them quickly, and the life cycle of the flies was the same as the life cycle of those fed cornmeal medium. The larval *D*. *hydei* consumed adult carcasses but were not found to consume puparia. We also observed that the feeding habits of *D*. *simulans* were similar to those of *D*. *melanogaster* (Fig. 5c,d, Movie S5); larval *D*. *simulans* consumed FAOs and adult carcasses, and combined cornmeal-FAO foods increas ed female fertility. It is interesting to note that female *D*. *sechellia*, which specialize on the toxic *Morinda* fruit, were also induced to lay large amount of eggs on Manila clam. Unfortunately, this species died out before we could perform further observations.

### *Drosophila* larvae digest food macromolecules externally

In the aqueous solutions collected from the food remains, the mean concentration of water-soluble protein (0.72 ± 0.17 mg/mL) was higher than that in the corresponding control substrates (0.60 ± 0.09 mg/mL); the mean concentrations of peptides and free amino acids were 0.52 ± 0.09 and 0.15 ± 0.03 mg/mL, respectively, and significantly higher (*t* = −3.744, P* = *0.02 and t = −4.932, P = 0.037, respectively) than those in the control foods (0.18 ± 0.02 and 0.06 ± 0.00 mg/mL, respectively). Many of the tiny scraps of meat fell off the food remains when they were rinsed, suggesting that they were hydrolyzed. The hard fat of the pork seemed rather palatable to *Drosophila* larvae. The mean free fatty acid concentration (0.10 ± 0.01 mg/mL) in the benzene solution collected from remains of the hard fat was higher than that of the control (0.08 ± 0.01 mg/mL). These results suggest that larvae egested digestive enzymes onto the foods that helped breakdown large food molecules and absorb the resulting nutrients.

**Figure 6:**
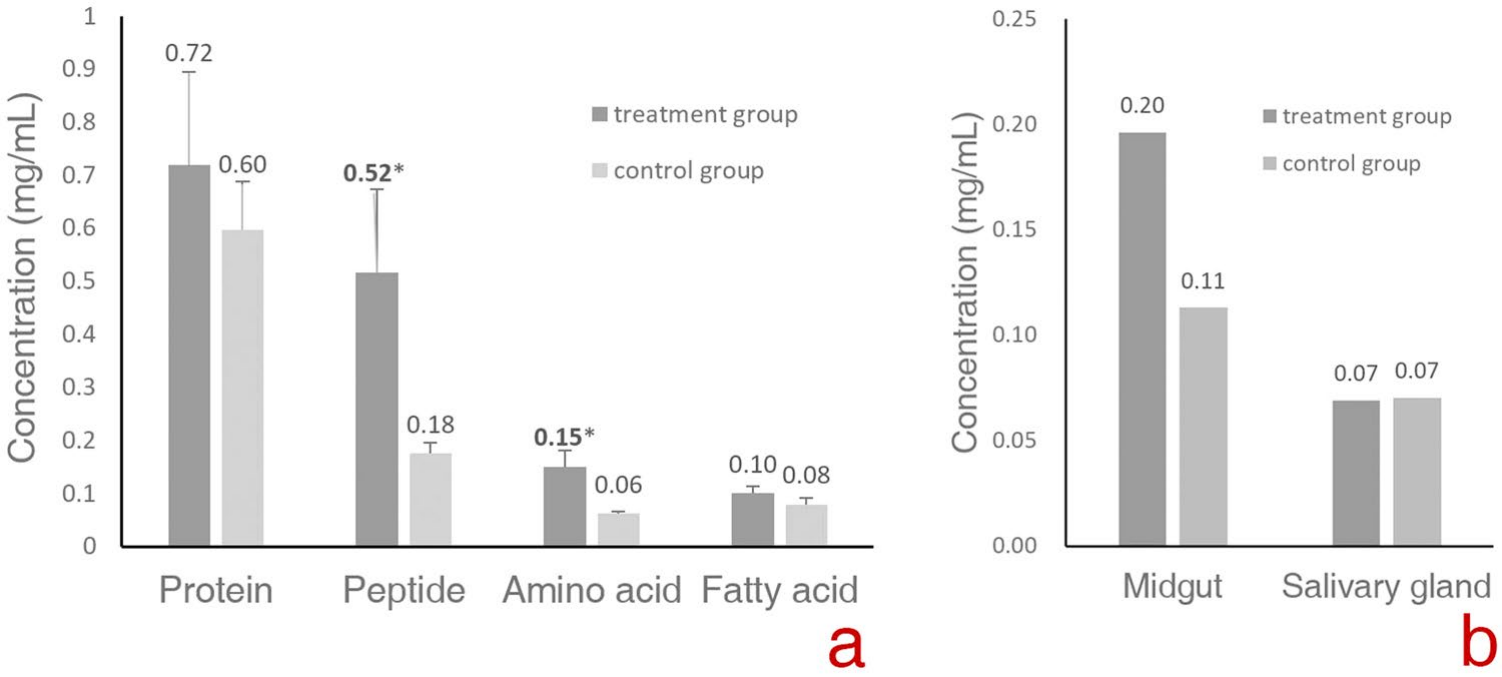
Macromolecule and amino acid concentrations in the solutions collected from the food remains (**a**) and amino acid concentrations in the digestive products of the gut and salivary gland homogenates (**b**).

It has been suggested that the digestive enzymes of *Drosophila* are produced by salivary glands^19^. To locate the organs responsible for producing digestive enzymes, gut and salivary gland homogenates were used to digest BSA. The concentrations of amino acids in the digestive products were determined. The differences in the concentrations of amino acids in the digestive products between the treatment group and control group were 0.08 mg/mL (guts) and 0.00 mg/mL (salivary glands). In the control group of the gut, the concentrations of amino acids were fairly high, and obviously, these amino acids came from FAOs (Fig. 6). These results indicated that the gut is the organ that makes proteinase.

## Discussion

Here, we show that *D*. *melanogaster* should be considered omnivorous. Larval *D*. *melanogaster* are able to meet their nutritional needs from microorganisms and plant/animal materials (including insect carcasses). Similar to *D*. *busckii*, a cosmopolitan flower-breeding species that breeds in animal-source garbage as well^10^, *D*. *melanogaster* can be breed out of a wide variety of FAOs without the help of microorganisms. Although adults cannot survive on animal diets, they choose and deposit eggs readily on them with or without the presence of yeasts. Obviously, the ability to exploit a wide range of foods helps this species cope with fluctuations in food availability and adapt to a diverse range of environmental conditions and allowed it to become cosmopolitan^23^. In addition to *D*. *melanogaster*, some other *Drosophila* species that belong to different subgroups are also omnivores, suggesting that there may be a good amount of omnivorous species in *Drosophila*.

Our results reveal that for *D*. *melanogaster*, a diet of the baker yeast *Saccharomyces cerevisiae* is better than FPO or FAO diets. In yeast foods, more eggs developed into adulthood, and the adults that emerged were larger in size and sound in body. A possible explanation may lie in the nutritional value of the foods. The calories (energy) in 100 g of baker yeast, beef, chicken (breast) and banana are 295, 187, 164, and 89, respectively^33^, and in the same amount of food, a larva can obtain much more energy for development from yeast. According to the optimal foraging theory^34–36^, *Drosophila* larvae forage in such a way as to maximize their energy intake per unit time and minimize developmental time^37^. Furthermore, the nutrition of yeast is more balanced^38,39^ than that of FPOs or FAOs. Dietary composition influences the life history characteristics of *Drosophila*^1,4,37^. The demand for both high-energy and nutritionally balanced food for development might drive flies such as *D*. *melanogaster* to adapt to and finally shift their food preference from original foods to yeasts, which have a natural association with fruits^40^.

Our results indicate that *Drosophila* larvae, similar to other animals that feed on several food sources to balance their nutritional requirements^41^, may feed on both yeast and FAOs and not on yeast alone when both foods are mixed together. The higher food quality and the nutrition-rich status of the combined diets may strongly affect egg-laying preference^42^ and egg production^43^ and ultimately significantly boost the fertility of flies. These results support the argument that the suitability of a medium for *Drosophila* is defined by the combination of yeast and specific substrates^8^. In fact, widely used cornmeal media, such as those described in^44^, perfectly demonstrate how *Drosophila* larvae successfully use both yeast and FPOs. However, as documented in other insects, such as grasshopper nymphs (*Schistocerca americana*)^45^ and the German cockroach (*Blattella germanica*)^46^, the foraging and egg-laying behaviors of adults and larvae are altered considerably when FAOs and yeast foods are placed apart. We will investigate these behaviors in future research.

Based on the above findings, we reconsider previous conclusions about the original adaptations of *Drosophila* and how they were subverted or broadened. Our results have direct implications for diet-related studies using *Drosophila* as a model organism and may help identify the set of digestive enzyme genes involved in FAO substrate digestion and their functions and further identify the impact of genetic changes in these gene on animal metabolism. These results might also help scientists to determine the exact nutrient requirements of the species of interest to create better recipes for laboratory culture of some flies^47^ or to design more effective control measures for some invasive pest species.

## Materials and Methods

### Fly stocks and handling of flies

The wild-type strain of *Drosophila melanogaster* was collected in Beijing, China, and kept in our laboratory for more than five years; *D*. *simulans* were kindly provided by Prof. Jian Lu (Peking University, Beijing, China). All flies used in this study were raised on cornmeal medium at 25 °C. The adult flies used in this study were 1–4-day-old individuals collected from corn meal cultures. The methods for transferring and anesthetizing adult flies were as described previously^48^ with slight modifications: the ice packs used here measured 26.5 cm × 14.5 cm × 2.5 cm and were covered by a piece of gauze to absorb the melted water. Adult flies were killed either by frost or by heating for 40 seconds in a 1300 Watt microwave oven.

Except where otherwise stated, in this paper, (1) the day when parental flies were placed into a culture vial is denoted as the 0^th^ day, and (2) means, standard deviations, etc. were calculated from the results of 3 replicates.

### Media and agar gel

*Cornmeal medium*: 105 g of cornmeal, 75 g of sucrose, 10 g of agar, 40 g of dry yeast, 6.25 ml of propionic acid, and 1.4 g of Nipagin per liter of distilled water. *Yeast medium*: 100 g of dry baker yeast *Saccharomyces cerevisiae*, 100 g of sugar, 8 g of agar, 1.42 g of Nipagin, and 6.52 ml of propionic acid per liter of distilled water. *Agar gel*: 20 g of agar; 6.25 ml of propionic acid, and 1.4 g of Nipagin per liter of distilled water. The internal dimension of the glass culture vials used here was 3 cm × 9 cm, and the amount of media or agar gel filled in was 10 ml.

### The FAOs

The FAOs used included fresh pork and beef, fresh/roasted chicken, boiled hen egg, and fresh meats of Manila clam *Ruditapes philippinarum*, Chinese razor clam *Sinonovacula constricta* and crucian carp *Carassius auratus*. These FAOs were all bought in a local supermarket. For larvae digestion analysis, 2 g of food was used; for the other analyses, 0.80 g of food and a cornmeal medium patch of the same weight were used as control food. The FAOs were rinsed with aseptic distilled water and air dried before being put into culture vials.

### Assay of quantifying egg-laying capacity and egg hatchability

The FAOs were placed on agar gel, and ten pairs of flies were introduced into each vial and allowed to oviposit for 72 hours at 25 °C. On the fifth day, the cumulative number of eggs (hatched plus unhatched eggs) was counted, and the proportion of hatched eggs was calculated to give a measure of hatchability.

### Assay of quantifying the ratio of adults emerged/100 eggs placed on different foods

*D*. *melanogaster* eggs were collected and placed into vials that contained 0.8 g of beef, pork, chicken, and clam (FAOs) and mango, banana, apple, peach, pear and muskmelon (FPOs), with 3 replications each. No chemical was added to these foods. Cornmeal medium was used as the control food. The adults that emerged from the FAOs and cornmeal medium were transferred into fresh cornmeal media, with 5 pairs in each vial and 2 replications. On the 7^th^ day, the parents were removed, and on the 15^th^ day, the number of progeny was counted. The sizes of the flies were measured by 30 females/males weighed in milligrams (mg). The sizes of their parents were not measured as the number of parents was quite lower.

### Assay of adult mortality and larvae development on exclusively FAOs

The FAOs were placed on 2% agar gel. Then, 10 pairs of adult *Drosophila melanogaster*/*D*. *virilis*/*D*. *hydei* were introduced into each vial. Mortality was recorded, and dead individuals were removed daily for 10 days. Some newly emerged adults were collected and killed, and their sizes were measured.

### Assay of larval food preference

The cornmeal-fed mixed-age larvae were rinsed with distilled water to remove any remaining food and then starved for 1 hour in agar (2%) Petri dishes. Then, groups of 30 larvae each were transferred with a fine moist paintbrush to the edge of an agar (2%) Petri dish (internal diameter 5.5 cm) approximately 2 cm away from one FAO and one cornmeal medium patch of equal weight and equal dimension (dimension 1.5 cm, 1 cm apart). The larvae were left to forage freely for 30 min, after which the larval aggregation in each food was quantified. Larvae that did not reach either of the food types were excluded from the analysis.

### Assay of the egg-laying preference of flies

One FAO and one cornmeal medium patch of equal weight and equal dimension (dimension 1.5 cm, 1 cm apart) were placed on agar (2%) gel filled in a 250-ml glass wide-mouth bottle (internal diameter 4.2 cm). Fifty females were placed into the bottle and were left to oviposit for 20 hours, after which each cornmeal medium patch and FAO were transferred to a new vial filled with agar gel. The eggs laid on the agar gel were discarded. Twenty-four hours later, the number of eggs (larvae and unhatched eggs) laid in each food was counted.

### Sterilization of eggs and FAOs

The surfaces of chicken and beef were sterilized with a 10% Clorox solution, and the flesh of the inner parts was cut with a pair of curved-tip surgical scissors and placed on autoclaved agar gel. Eggs were sterilized in a 10% Clorox solution for 8 min, rinsed three times with sterile distilled water^19^ and placed on sterile chicken and beef. All these processes were carried out on a superclean bench.

### Assay of digestion products and proteinase-producing organ

*For the protein*, *peptide and amino acids assay*, two hundred mix-aged *D*. *melanogaster* larvae were placed on 2 g of chicken and allowed to forage freely for 12 hours; then, the larvae were removed, and the remaining food was rinsed 5 times with 5 ml of distilled water. The resulting solution was collected and centrifuged at 3500 rpm for 10 min, and the supernatant was collected. *For the protein assay*, 0.5 ml of supernatant was added, and 0.5 ml of distilled water was added to 3 ml of Coomassie brilliant blue^49^; these were mixed and incubated for 10 min, and the absorbance was measured at 595 nm. *For the peptide assay*, 0.75 ml of 10% (w/v) trichloroacetic acid (TCA) was added to the same volume of supernatant; this was mixed thoroughly, incubated for 15 min, and then centrifuged at 3500 rpm at 10 min, and the supernatant was collected. Of this supernatant, 1.4 ml was added to 2 ml of Biuret reagent^50^, and the absorbance was read at 540 nm. *For the amino acids assay*, 0.75 ml of 10% TCA was added to the same volume of supernatant and centrifuged at 3500 rpm for 10 min, and 1 ml of supernatant was added along with 1 ml of sodium acetate buffer (pH 5) to 2 ml 2% (w/v) aqueous solution of ninhydrin and incubated in a 100 °C water bath for 19 min. The absorbance was read at 568 nm. *For the fatty acids assay*, two hundred larvae were placed on 2 g of hard pork fat and left to forage for 12 hours. The food that remained was rinsed 5 times with 5 ml of benzene. The resulting solution was collected and centrifuged at 3500 rpm for 10 min. Then, 3 ml of supernatant was added to 2 ml of 5% (w/v) aqueous solution of cupric acetate^51^, shaken thoroughly, and incubated for 10 min, and the upper layer was read at 715 nm. *To locate the proteinase-producing organ*, large and easy-to-dissect *D*. *hydei* larvae were fed chicken for 12 hours and then dissected in distilled water. Intact salivary glands and guts (severed at the points of foregut imaginal ring and hindgut imaginal ring) were collected from approximately 100 larvae, and these parts were homogenized in 0.5 ml of distilled water. The treatment group had 0.2 ml of homogenate, 0.2 ml of phosphate buffer (pH 6.0), and 0.2 ml of 1 mg/ml BSA. The control group had the same volume of 10% TCA added to the solution, whose solute was the same as the treatment group, and incubated in a 37 °C water bath for 30 min. Then, the same volume of 10% TCA was added to the solution of the treatment group, and it was mixed and incubated at room temperature for 15 min. Then, amino acid analysis was performed as described above.

### Photography and video recording

The behaviors were photographed and video recorded using a Nikon Coolpix P330 compact camera mounted to an eyepiece of the stereo microscope via an adapter. The photographs and videos were edited using Adobe^®^ Photoshop and Windows Movie Maker, respectively. A transparent 52-mm ultraviolet (UV) filter was used as needed to cover the vials to prevent the adult flies from escaping.

### Statistical analysis

All statistical analyses were conducted using IBM SPSS Statistics for Windows, Version 22.0, Armonk, NY: IBM Corp^52^. The significances of the difference in the means from the mean of the corresponding control group were determined by one-way ANOVA followed by Dunnett’s test for multiple comparison. Comparisons between the cumulative survival rates of the adults maintained in agar gel and animal diets were performed using the Kruskal-Wallis *H*-test, and comparisons between the cumulative survival rates of adults kept in cornmeal medium and FAO culture were performed using Mann-Whitney U test^53^.

## Electronic supplementary material

The legends for supplementary videos

Drosophila melanogaster feed on beef

Drosophila melanogaster feed on chicken

D.melanogaster larvae eat carcass

D.melanogaster larvae eat carcass in cornmeal

D.simulans eat beef

## References

[1] Ormerod KG (2017). *Drosophila* development, physiology, behavior, and lifespan are influenced by altered dietary composition. Fly.

[2] Plesnar-Bielak A (2017). Larval and adult nutrition effects on reproductive traits in the red flour beetle. J. Zool..

[3] Lachaise D (1988). Historical biogeography of the *Drosophila-melanogaster* species subgroup. Evol. Biol..

[4] Simpson, S. J. & Raubenheimer, D. *The Nature of Nutrition: A Unifying Framework from Animal Adaptation to Human Obesity*. (Princeton University Press: Princeton, 2012.)

[5] Ryuda M, Tsuzuki S, Tanimura T, Tojo S, Hayakawa Y (2008). A gene involved in the food preferences of larval *Drosophila melanogaster*. J. Insect. Physiol..

[6] Dworkin I, Jones CD (2009). Genetic Changes Accompanying the Evolution of Host Specialization in *Drosophila sechellia*. Genetics.

[7] Jin K (2011). Why Does the Giant Panda Eat Bamboo? A Comparative Analysis of Appetite-Reward-Related Genes among Mammals. Plos One.

[8] Begon, M. Yeasts and *Drosophila*. In *Genetics and Biology of Drosophila*, vol. 3b (Eds Ashburner, M., Carson, H. L. & Thompson, J. N.) 345–384 (Academic Press Inc. London, 1982).

[9] Staats Stefanie, Wagner Anika, Kowalewski Bianca, Rieck Florian, Soukup Sebastian, Kulling Sabine, Rimbach Gerald (2018). Dietary Resveratrol Does Not Affect Life Span, Body Composition, Stress Response, and Longevity-Related Gene Expression in Drosophila melanogaster. International Journal of Molecular Sciences.

[10] Sturtevant, A. H. *The North American species of Drosophila*. (Carnegie Institution of Washington: Washington, 1921).

[11] Klaczko LB, Powell JR, Taylor CE (1983). *Drosophila* baits and yeasts - species attracted. Oecologia.

[12] Becher PG (2012). Yeast, not fruit volatiles mediate *Drosophila melanogaster* attraction, oviposition and development. Funct. Ecol..

[13] Carson, H. L. The ecology of *Drosophila* breeding sites. In *Harold L Lyon Arboretum Lecture*, vol. 2 (Ed. Harold, L.) 1–27 (The University of Hawaii, Honolulu, 1971).

[14] Cooper DM (1960). Food preferences of larval and adult *Drosophila*. Evolution.

[15] Stensmyr MC (2012). A Conserved Dedicated Olfactory Circuit for Detecting Harmful Microbes in *Drosophila*. Cell.

[16] Masek Pavel, Keene Alex C. (2013). Drosophila Fatty Acid Taste Signals through the PLC Pathway in Sugar-Sensing Neurons. PLoS Genetics.

[17] Montell C (2009). A taste of the *Drosophila* gustatory receptors. Curr. Opin. Neurobiol..

[18] Dombrovski M (2017). Cooperative Behavior Emerges among *Drosophila* Larvae. Curr. Biol..

[19] Gregg TG, McCrate A, Reveal G, Hall S, Rypstra AL (1990). Insectivory and social digestion in *Drosophila*. Biochem. Genet..

[20] Lemaitre B, Miguel-Aliaga I (2013). The Digestive Tract of *Drosophila melanogaster*. Annu Rev Genet.

[21] Pereira HS, Sokolowski MB (1993). Mutations in the larval foraging gene affect adult locomotory behavior after feeding in *Drosophila melanogaster*. Proc. Natl. Acad. Sci..

[22] Caldwell JC, Miller MM, Wing S, Soll DR, Eberl DF (2003). Dynamic analysis of larval locomotion in *Drosophila* chordotonal organ mutants. Proc. Natl. Acad. Sci..

[23] Keller A (2007). *Drosophila melanogaster*’s history as a human commensal. Curr. Biol..

[24] Aydin E, Uysal S, Akkuzu B, Can F (2006). Nasal myiasis by fruit fly larvae: a case report. Eur. Arch. Oto-Rhino-L..

[25] Ahmad M, Chaudhary SU, Afzal AJ, Tariq M (2015). Starvation-Induced Dietary Behaviour in *Drosophila melanogaster* Larvae and Adults. Sci. Rep..

[26] Rypstra AL, Gregg TG (1986). Facultative carnivory in *Drosophila hydei*. DIS.

[27] Vijendravarma RK, Narasimha S, Kawecki TJ (2013). Predatory cannibalism in *Drosophila melanogaster* larvae. Nat. Commun..

[28] Dugatkin, L. A. Antipredator Behavior. In *Principles of animal behavior*, 3 edn. 383–415 (W. W. Norton & company: New york, London, 2014).

[29] Waldbauer GP, Cohen RW, Friedman S (1984). Self-Selection of an Optimal Nutrient Mix from Defined Diets by Larvae of the Corn Earworm, *Heliothis zea* (Boddie). Physiol. Zool..

[30] Waldbauer GP, Friedman S (1991). Self-selection of optimal diets by insects. Annu. Rev. Entomol..

[31] Richardson ML, Mitchell RF, Reagel PF, Hanks LM (2010). Causes and Consequences of Cannibalism in Noncarnivorous Insects. Annu. Rev. Entomol..

[32] Piper MDW (2015). A holidic medium for *Drosophila melanogaster*. Nat. Methods..

[33] Foods. https://www.fatsecret.com/calories-nutrition/.

[34] Pyke GH, Pulliam HR, Charnov EL (1977). Optimal foraging - selective review of theory and tests. Q. Rev. Biol..

[35] Smith JM (1978). Optimization theory in evolution. Annu. Rev. Ecol. Syst..

[36] Krebs, J. R. *Optimal foraging: decision rules for predators*. In *Behavioural ecology an evolutionary approach*. (Eds Krebs, J. R. & Davies, N. B.) 23–63 (Blackwell Scientific Publications, 1978).

[37] Rodrigues MA (2015). *Drosophila melanogaster* larvae make nutritional choices that minimize developmental time. J. Insect. Physiol..

[38] Nutritional Yeast: The Antiviral, Antibacterial Immune-Booster. https://draxe.com/nutritional-yeast/.

[39] Sang, J. H. *The nutritional requirements of Drosophila*. In *The genetics and biology of Drosophila*. Vol 2a.(Eds Ashburner, M. & Wright,T. R. F.) 159–192 (Academic Press Inc., London & New York, 1978).

[40] Fleet, G. H. Yeasts in fruit and fruit products. in *Yeasts in Food* (Eds Boekhout, T. & Robert, V.) 267–287 (Woodhead Publishing, 2003).

[41] Jensen K (2012). Optimal foraging for specific nutrients in predatory beetles. Proc. Roy. Soc. B-Biol. Sci..

[42] Betti MIL, Soto EM, Hasson E (2014). Oviposition Site Preference for Natural Breeding Sites in *Drosophila melanogaster* (Diptera: Drosophilidae) Populations From Argentina. Ann. Entomol. Soc. Am..

[43] Terashima J, Bownes M (2004). Translating available food into the number of eggs laid by *Drosphila melanogaster*. Genetics.

[44] Ashburner, M. & Thompson, J. N. Jr. The laboratory culture of *Drosophila*. In *The genetics and biology of Drosophila* Vol *2a* (Eds Ashburner, M. & Wright, T. R. F.) 1–109 (Academic Press: London, 1978).

[45] Bernays EA, Angel JE, Augner M (1997). Foraging by a generalist grasshopper: The distance between food resources influences diet mixing and growth rate (Orthoptera: Acrididae). J. Insect. Behav..

[46] Ko AE, Jensen K, Schal C, Silverman J (2017). Effects of foraging distance on macronutrient balancing and performance in the German cockroach *Blattella germanica*. J. Exp. Biol..

[47] Markow Therese A., O'Grady Patrick M. (2006). Dietary considerations. Drosophila.

[48] Qu W-h, Zhu T-b, Yang D-X (2015). A Modified Cooling Method and its Application in *Drosophila* Experiments. J. Biol. Educ..

[49] Bradford MM (1976). Rapid and sensitive method for quantitation of microgram quantities of protein utilizing principle of protein-dye binding. Anal. Biochem..

[50] Biuret Protein Assay. http://www.ruf.rice.edu/~bioslabs/methods/protein/biuret.html.

[51] Lowry RR, Tinsley IJ (1976). Rapid colorimetric determination of free fatty-acids. J. Am. Oil. Chem. Soc..

[52] Meyers, L. S., Gamst, G. C. & Guarino, A. J. *Performing data analysis using IBM SPSS®*. (John Wiley & Sons, Inc. Hoboken, New Jersey, 2013).

[53] Lee Elisa T., Wang John Wenyu (2003). Statistical Methods for Survival Data Analysis.

