# Supplementary material for: Carnivory in the larvae of Drosophila melanogaster and other Drosophila species
Source: Sci Rep. 2018 Oct 19;8:15484. doi: 10.1038/s41598-018-33906-w (PMC6195549; doi:10.1038/s41598-018-33906-w)
Supplement: Supplementary file 1 — The legends for supplementary videos [file 41598_2018_33906_MOESM1_ESM.docx]

# Carnivory in the larvae of *Drosophila* *melanogaster* and other *Drosophila* species

Daxiang Yang

Department of Zoology and Animal Physiology, College of Biological Sciences, China Agricultural University, 100193, Beijing, China

**The legends for supplementary videos**

**Movie S1**

Larval and adult *Drosophila melanogaster* feed on beef placed on cornmeal medium

**Movie S2**

*Drosophila melanogaster* larvae consume chicken placed on live yeast medium

**Movie S3**

*Drosophila melanogaster* larvae consume adult carcassed in a Manila clam culture.

**Movie S4**

*Drosophila melanogaster* larva consume adult carcass in cornmeal medium

**Movie S5**

*Drosophila simulans* larvae consume beef placed on cornmeal medium
